# Seasonal Chemical Variability and Antimicrobial, Anti-Proliferative Potential of Essential Oils from *Baccharis uncinella*, *B. retusa*, and *B. calvescens* (Asteraceae)

**DOI:** 10.3390/plants14091311

**Published:** 2025-04-26

**Authors:** Tânia F. Dlugoviet, Aurea P. Ferriani, Ana Paula P. Klein Hendges, Rebeca G. Camargo, Marta C. T. Duarte, Renata M. T. Duarte, Ana Lúcia Tasca Gois Ruiz, Noemi Nagata, Francisco A. Marques, Beatriz H. L. N. Sales Maia

**Affiliations:** 1 Departamento de Química, Universidade Federal do Paraná, Curitiba 81531-980, PR, Brazil; taniadlugoviet@gmail.com (T.F.D.); aurea.portes@hotmail.com (A.P.F.); rebecadecamargo@gmail.com (R.G.C.); nnagata@ufpr.br (N.N.); tic@ufpr.br (F.A.M.); 2 Central de Análises, Universidade Tecnológica Federal do Paraná, Pato Branco 85503-390, PR, Brazil; anakleinhendges@gmail.com; 3 Divisão de Microbiologia, CPQBA, Universidade Estadual de Campinas, Campinas 13148-218, SP, Brazil; mduarte@cpqba.unicamp.br (M.C.T.D.); duartemt@unicamp.br (R.M.T.D.); 4 FCF, Universidade Estadual de Campinas, Campinas 13083-871, SP, Brazil; ana.ruiz@fcf.unicamp.br

**Keywords:** *Baccharis*, seasonal variation, anti-proliferative activity, antimicrobial activity

## Abstract

Essential oils (EOs) of three native species *Baccharis uncinella*, *B. retusa* and *B. calvescens*, obtained through hydrodistillation, were analyzed by GC-MS and GC-FID for seasonality, and the antimicrobial and anti-proliferative activities were evaluated. EO of *B. calvescens* and *B. uncinella* consisted mainly of oxygenated sesquiterpenes, while in the EO of *B. retusa*, monoterpene hydrocarbons were predominant. The highest antimicrobial activity was observed for spring *B. uncinella* EO against *S. aureus*, *C. albicans* and summer *B. uncinella* EO against *C. albicans* and *B. subtilis*. Essential oils of *B. calvescens* showed more effective anti-proliferative activity than *B. retusa* EO and *B. uncinella* EO. This is the first study of the EO of *B. retusa*, and it was demonstrated that the majority composition was different in all seasons of the year, justifying the importance of the seasonal study. Furthermore, the summer and spring EO showed potent cytostatic effects against the K562 and OVCAR-03 cell lines, respectively. For each species, PCA differentiated the EO chemical composition seasonally. PCA of all samples distinguished the three species. This study underscores the importance of assessing seasonal variation in the chemical composition and biological activities of essential oils, highlighting the potential of compounds spathulenol, caryophyllene oxide, limonene and α-pinene for achieving the desired product properties.

## 1. Introduction

The genus *Baccharis*, which belongs to the Asteraceae family, includes about 500 species commonly known as brooms. They are found mainly in South America, and in countries such as Brazil, Argentina, Colombia, Chile, and Mexico [1,2]. Several species are used in folk medicine for the treatment of anemia, inflammation, diabetes, and diseases of the stomach, liver, and prostate, among other ailments. Antimicrobial, insect repellent, insecticide, anti-ulcerogenic, cytotoxic, anti-inflammatory, and sedative properties have also been described [3].

The essential oils (EOs) of approximately 50 species of *Baccharis* have been studied for chemical composition [4]. In general, they are characterized by the presence of cyclic alcohols of sesquiterpenes (spathulenol, globulol, palustrol, ledol and viridiflorol) [5]. Additionally, the presence of germacrene D, bicyclogermacrene and caryophyllene oxide has also been highlighted [6].

Most of the studied species were collected in Brazil, mainly in the South region [7], and when evaluated for biological activity, they presented important results. Among the EO of the species studied, *B. salicifolia*, for example, presented antimicrobial, toxic and repellent effects [8]. *Baccharis coridifolia* demonstrated antibacterial and antibiotic-modulating activities, showing potential in controlling *Escherichia coli*, *Pseudomonas aeruginosa* and *Staphylococcus aureus*, acting against bacterial resistance [9].

*Baccharis darwinii* exhibited insecticidal and repellent actions, as well as antifungal activity [10]. *Baccharis uncinella* showed antimicrobial properties [11] and a sedative effect whose intensity is associated with the ratio of mono/sesquiterpenes compounds present in the sample, a proportion that can be affected by ecological factors related to plant protection [12]. Although genotypic characteristics and phenology of individuals or species determine their main phytochemical characteristics, biotic conditions (pathogens, plant-pathogen interactions, symbiotic strategies) [13,14], abiotic factors (light, temperature, soil composition and water availability) and geographic conditions may exert a strong influence on the production of secondary metabolites [15,16].

Seasonality influences the expression of volatile compounds in plants, affecting their biosynthesis, storage, and emission, especially due to reproductive/vegetative life cycles and stress conditions. Temporal variation in phytochemicals throughout the day and year is observed in diverse plant species. Understanding this temporal control can improve the production of selected phytochemicals, with careful consideration of harvest timing when selecting plants for enhanced phytochemical activity. Additionally, temporal and environmental variation can be used to identify regulatory and biosynthetic pathways, optimizing efforts to increase the production of desired compounds [17,18].

Despite this, there is limited research on the impact of seasonality on volatile chemical profiles in *Baccharis* L. [2,6,15,17,19,20]. Further studies are needed to examine how the chemical composition of volatile compounds varies throughout the year. This is especially important for aromatic species that have high economic value.

In the present study, the EOs of three native *Baccharis* species (*B. uncinella*, *B. retusa* and *B. calvescens*) were analyzed seasonally to expand the knowledge about EOs and identify possible chemotaxonomic markers for the species. *B. uncinella* EO has already been studied by several authors [4,12,21,22,23,24,25], and some studies indicate that there are chemical variations in the production of secondary metabolites depending on the period and place of collection [6,12,25,26], which can affect their biological properties [12]. Thus, the study seeks to contribute to evaluating the seasonal effect on the chemical composition of the EOs and their antimicrobial and anti-proliferative activities of species collected in the southern region of Brazil. To our knowledge, this constitutes the first report of how seasonality affects the chemical composition of the essential oil of *B. retusa* and *B. calvescens*.

## 2. Results and Discussion

### 2.1. Chemical Composition

The EO samples were obtained by hydrodistillation of *B. uncinella*, *B. retusa* and *B. calvescens* dried leaves collected in each season of the year. The yields, calculated based on the weight of dry leaves, were determined to be 0.05–0.29%, with the highest yields being obtained for *B. retusa* in winter. One hundred and thirty-eight compounds were identified among EO samples from *B. uncinella*, *B. retusa* and *B. calvescens* by GC-MS and GC-FID (Table 1).

The main compounds present in *B. calvescens* EO were spathulenol (2.86% to 14.65%), caryophyllene oxide (3.17% to 11.94%), germacrene D (1.41% to 6.58%), α-cadinol (0 to 6.38%), (*E*)-caryophyllene (0 to 5.96%) and humulene epoxide II (1.41% to 5.69%). Spathulenol is the major compound in all collection seasons of *B. calvescens* leaves, except in the autumn sample, where germacrene D stands out as the major compound.

*B. retusa* EO were mainly composed of limonene (2.51% to 11.90%), germacrene D (0 to 11.74%), α-pinene (5.18% to 11.16%), spathulenol (4.33% to 10.27%), (*E*)-caryophyllene (0 to 9.05%), α-thujene (4.10% to 8.71%), bicyclogermacrene (0 to 8.30%), caryophyllene oxide (0 to 6.73%), γ-curcumene (0 to 6.11%), β-pinene (2.73% to 5.88%), γ-muurolene (0 to 5.41%), dauca-5,8-diene (0 to 5.04%) and (*Z*)-caryophyllene (0 to 5.03%).

*Baccharis uncinella* EO was majorly composed of spathulenol (11.06% to 15.09%), caryophyllene oxide (0 to 6.63%), α-pinene (2.47% to 6.08%), limonene (3.94% to 5.83%) and globulol (0 to 5.63%).

### 2.2. Principal Component Analysis (PCA)

PCA of *B. calvescens* EO generated three Principal Components (PCs), of which Principal Component 1 (PC1) explained 39.06% of the total variance, while Principal Component 2 (PC2) and Principal Component 3 (PC3) explained 26.30% and 16.82%, respectively.

**Figure 1:**
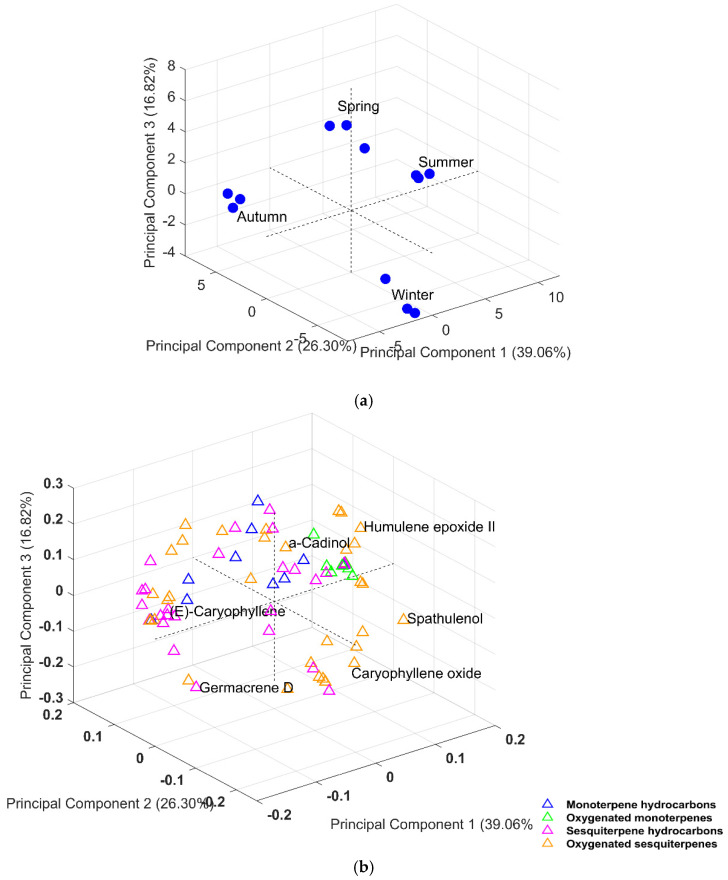
The plot of scores (**a**) and loadings (**b**) from the Principal Component Analysis of essential oils from leaves of *Baccharis calvescens*. PCA explained 82.18% of the total variance of the data (PC1: 39.06%, PC2: 26.30% and PC3: 16.82%).

Scores of the three PCs differentiated the samples from each season (Figure 1a). Oxygenated sesquiterpenes were predominant in all seasons EO (45.25% to 47.21%) except in the autumn sample, in which sesquiterpene hydrocarbon was the main class (42.22%). Loadings (Figure 1b) have shown that, in this EO, sesquiterpene hydrocarbons germacrene D (6.58%), bicyclogermacrene (4.68%), and β-elemene (4.24%) were present in higher concentrations than the others. Furthermore, sesquiterpene hydrocarbons *trans*-cadina-1(6)-diene (1.24%) and germacrene A (1.18%) were found only in the autumn season.

Winter and spring sample class concentrations were very similar, but they could be differentiated based on the presence of specific metabolites. There were a few compounds found only in the winter EO, such as sesquiterpene hydrocarbon (*Z*)-γ-bisabolene (1.23%) as well as oxygenated sesquiterpenes *cis*-cadin-4-en-7-ol (1.86%) and amorphan-4,9-dien-2-ol (0.91%). In addition, caryophyllene oxide was present in the highest concentration in this season (11.94%). As for the spring sample, sesquiterpene hydrocarbon β-calacorene (0.43%), oxygenated sesquiterpenes α-cadinol (6.38%), iso-bicyclogermacrenal (0.68%) and β-acoradienol (0.46%) were only found in this season.

Monoterpene hydrocarbons and oxygenated monoterpenes were present in the highest amounts in the summer season (26.16% overall). The monoterpene hydrocarbon α-pinene was present in higher concentrations in this EO (4.20%), while oxygenated monoterpenes *trans*-verbenol (1.09%), pinocarvone (1.31%) and myrtenol (2.17%) were found only in this season. Therefore, this was the best collection period to obtain higher levels of these compounds.

In summer, long days associated with high temperatures appear to favor monoterpene biosynthesis [27,28]. Studies with other *Baccharis* species also showed variation in chemical composition depending on seasonality [6].

PCA of *B. retusa* EO generated three PCs, of which PC1 explained 35.25% of the total variance, while PC2 and PC3 explained 26.76% and 22.34%, respectively.

**Figure 2:**
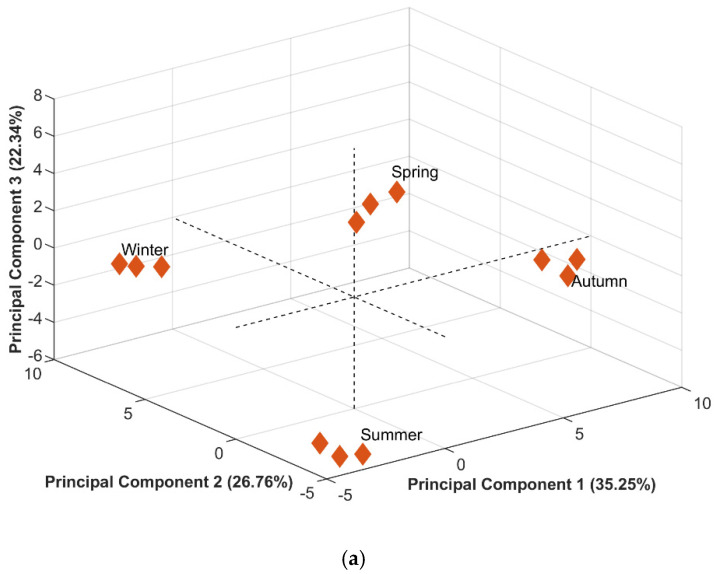

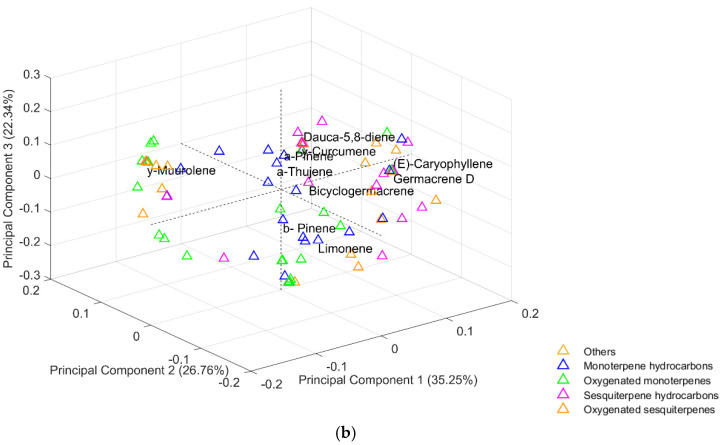
The plot of scores (**a**) and loadings (**b**) from the Principal Component Analysis of essential oils from leaves of *Baccharis retusa*. PCA explained 84.35% of the total variance of the data (PC1: 35.25%, PC2: 27.76% and PC3: 22.34%).

Scores of the three PCs differentiated the samples from each season (Figure 2a). Sesquiterpene hydrocarbon amounts were higher in the autumn EO (41.71%), which differentiated this sample from the others. Loadings (Figure 2b) have shown (*E*)-caryophyllene (9.05%) and germacrene D (11.74%) as major compounds only found in this season. Other major compounds were bicyclogermacrene (8.30%) and monoterpene hydrocarbon α-pinene (5.18%). In addition, oxygenated sesquiterpenes viridiflorol (1.84%) and rosifoliol (1.06%) were only found in this sample.

Monoterpene hydrocarbons were the main class present in the spring and summer EO (43.33% and 40.96%, respectively), with α-pinene (11.16% and 5.53%, respectively), limonene (6.38% and 11.90%, respectively) and β-pinene (5.88% and 5.61%, respectively) as major compounds. On the other hand, the summer season showed the highest amounts of oxygenated monoterpenes, with cryptone (1.73%) and *p*-menth-1-en-7-al (1.42%) present only in this sample. As for the spring sample, it was majorly composed of α-thujene (8.71%), as well as sesquiterpene hydrocarbons γ-curcumene (6.11%), dauca-5,8-diene (5.04%) and (*Z*)-caryophyllene (5.03%).

The winter season EO showed similar quantities of monoterpene hydrocarbons, sesquiterpene hydrocarbons and oxygenated sesquiterpenes (around 23%). Sesquiterpene hydrocarbon γ-muurolene (5.41%) was found only in this season. Other major compounds were monoterpene hydrocarbon α-pinene (5.83%), sesquiterpene hydrocarbons bicyclogermacrene (5.32%) and (*Z*)-caryophyllene (5.03%) and oxygenated sesquiterpenes spathulenol (10.27%) and caryophyllene oxide (6.73%).

Seasonality has a great influence on the yield and chemical composition of essential oils. Climatic factors specific to each season of the year, such as radiation and precipitation, can influence the production of volatile compounds in plants. In studies with *B. dracunculifolia*, the concentration of spathulenol increased approximately three times in autumn compared to winter [29]. On the other hand, our study with *B. retusa*, the concentration of spathulenol was much higher in winter (10.27%) than in autumn (4.33%).

To our knowledge, this is the first study on *B. retusa* essential oil. Previous reports on the chemical composition of crude extracts from this species [30,31] include γ-cadinene, caryophyllene oxide and humulene oxide in the n-hexane extract of its aerial parts. Notably, caryophyllene oxide was identified as a major compound in the EO of this species in our study.

PCA of *B. uncinella* EO generated two PCs, of which PC1 explained 35.93% and PC2 explained 29.72% of the total variance.

**Figure 3:**
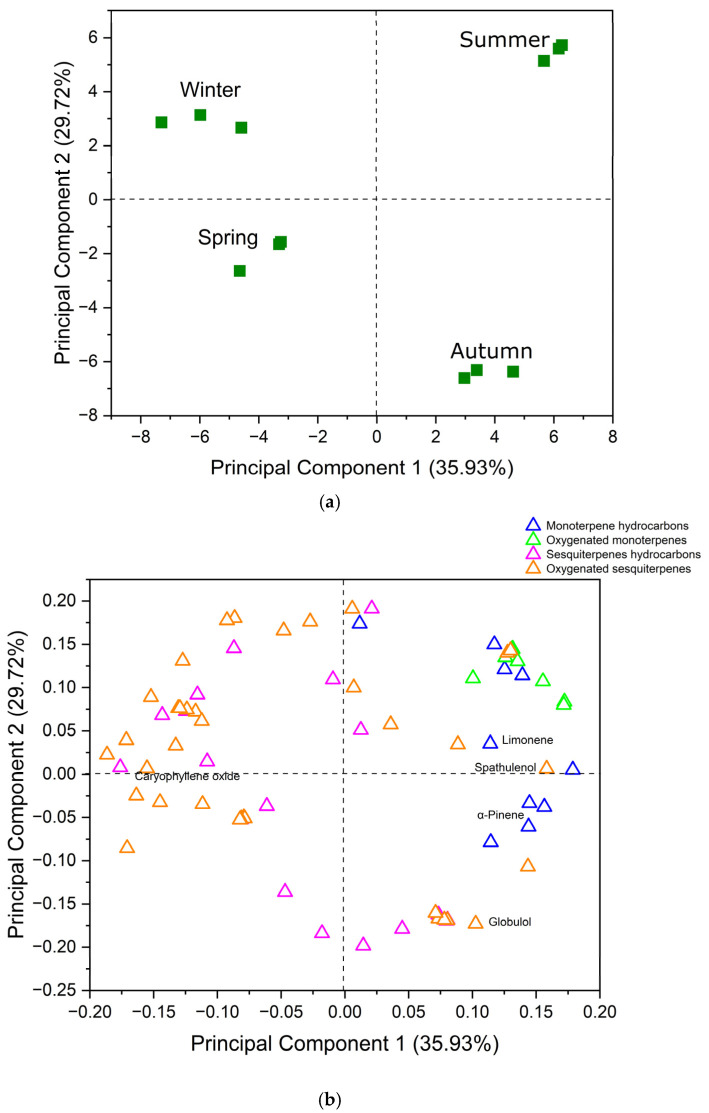
The plot of scores (**a**) and loadings (**b**) from the Principal Component Analysis of essential oils from leaves of *Baccharis uncinella*. PCA explained 65.65% of the total variance of the data (PC1: 35.93% and PC2: 29.72%).

Scores of the two PCs differentiated the samples from each season (Figure 3). PC1 differentiated the winter and spring from the summer and autumn EO. This could be explained by the higher amounts of oxygenated sesquiterpenes in the winter and spring samples (53.94% and 50.84%, respectively), with caryophyllene oxide as a major compound present only in these EO (5.00% and 6.63%, respectively). On the other hand, the summer and autumn samples showed monoterpene hydrocarbon α-pinene as a major compound (6.08% and 5.81%, respectively).

Ascari [12] also verified spathulenol and caryophyllene oxide as the main components in EO from *B. uncinella* collected in Santa Catarina, similar composition to our study.

In studies with leaves of *B. dracunculifolia*, multivariate analyses grouped the summer and autumn seasons with high levels of (*E*)-nerolidol and winter and spring with high levels of β-pinene, α-pinene and germacrene D. Emphasizing that biotic and abiotic factors specific to each season affect the quantitative variation in volatile compounds [29].

In our study, differences in the presence of specific metabolites could be observed in each season EO. The winter sample was differentiated mainly by oxygenated sesquiterpenes, such as salvial-4(14)-en-1-one (2.15%), shyobunol (1.70%), cubenol (1.47%) and 10-*epi*-γ-eudesmol (1.08%), which were present only in this EO. In addition, *epi*-α-muurolol (4.22%), germacra-4(15),5,10(14)-trien-1α-ol (3.26%) and humulene epoxide II (3.25%) were present in higher amounts in this sample.

The spring EO could be differentiated by sesquiterpene hydrocarbon *cis*-muurola-4(14),5-diene (1.42%) and oxygenated sesquiterpenes *cis*-cadin-4-en-7-ol (2.75%), *cis*-dihydro-mayurone (1.54%), 1,10-di-*epi*-cubenol (1.45%) and thrujopsan-2α-ol (1.29%), which were only present in this season sample.

In the summer EO, monoterpene hydrocarbons and oxygenated monoterpenes were present in higher concentrations (33.14% overall). Limonene (5.83%) was a major compound and terpinen-4-ol (2.92%) was present in higher amounts than in the other samples.

Spathulenol is present in large quantities in all collection seasons, being higher in summer and lower in spring. According to [20], in a study with *B. dracunculifolia*, the average concentration of spathulenol increased approximately three times from July (16.25 mg/100 g of the plant) to April (47.50 mg/100 g of the plant), being higher in February (51.21 mg/100 g of the plant). In another study, with *B. trimera*, the concentration of spathulenol was higher in March (12%) [6].

The autumn EO was differentiated by the higher concentrations of sesquiterpene hydrocarbons α-muurolene (2.78%) and (*E*)-caryophyllene (4.57%), the latter being present only in this sample. In addition, oxygenated sesquiterpenes globulol (5.63%), *allo*-aromadendrene epoxide (3.50%) were present in higher amounts, whilst guaia-3,10(14)-dien-11-ol (1.06%) was present only in this EO.

It is observed that for the three species studied, the composition of the essential oil can change throughout the year; that is, the content of some major compounds can vary with the seasonality. Thus, knowing this variation helps to determine the best time of harvest to obtain a specific compound, improving the quality of the raw material.

PCA of the three species EO generated 11 PCs, of which PC1 explained 20.85% and PC2 explained 13.09% of the total variance.

**Figure 4:**
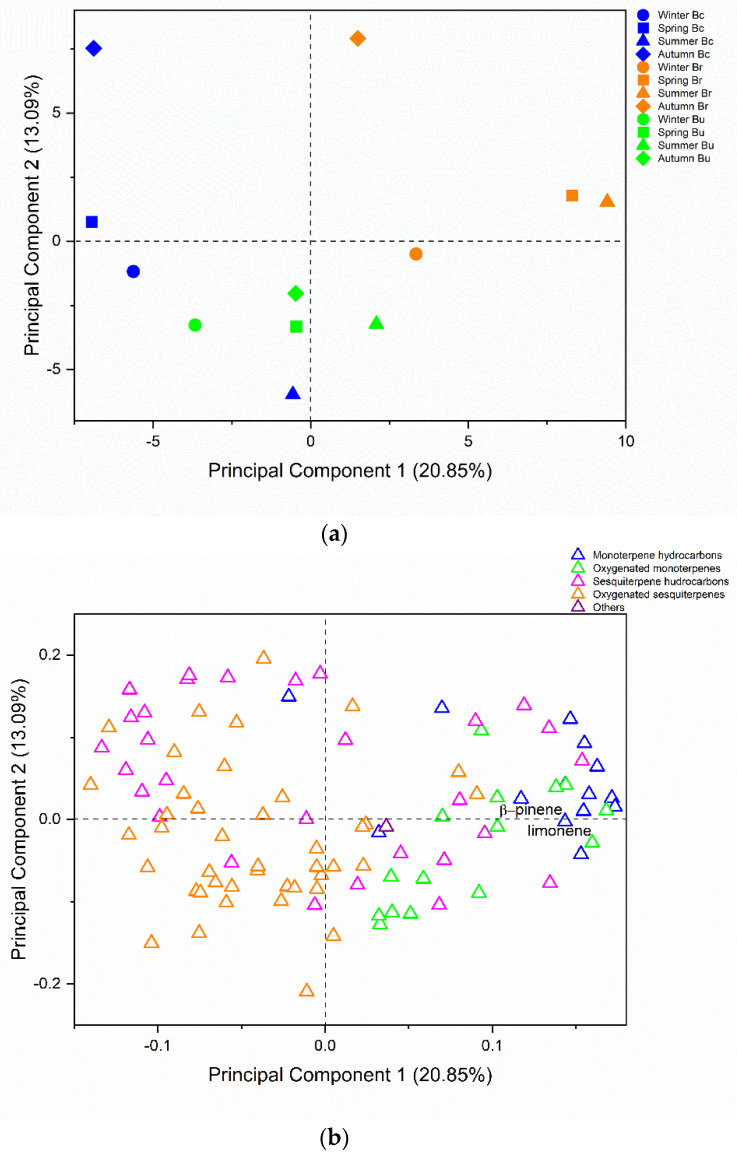
The plot of scores (**a**) and loadings (**b**) from the Principal Component Analysis of essential oils from leaves of *Baccharis calvescens*. *B. reutsa* and *B. uncinella*. PCA explained 33.94% of the total variance of the data (PC1: 20.85% and PC2: 13.09%).

Scores of PC1 and PC2 could differentiate the samples based on compound classes (Figure 4). Winter, spring and summer samples from *B. calvescens* and winter, autumn and spring samples from *B. uncinella* were grouped based on their high contents of oxygenated sesquiterpenes (45.25 to 53.94%).

Spring and summer EO from *B. retusa* showed the highest concentrations of monoterpene hydrocarbons amongst all samples (40.96% to 43.33%), especially β-pinene and limonene, major compounds in these samples.

In addition, the winter sample from *B. retusa* and the summer sample from *B. uncinella* showed similar amounts of monoterpene hydrocarbons and oxygenated monoterpenes (25.65% and 9.63% versus 24.36% and 8.78%, respectively).

In another study on the chemical composition of *Baccharis* species (*Baccharis anomala*, *B. calvescens*, *B. mesoneura*, *B. millefora*, *B. oblongifolia*, *B. trimera* and *B. uncinella*), large amounts of monoterpene hydrocarbons (α-thujene, α-pinene, β-pinene and limonene) and oxygenated sesquiterpenes (globulol and spathulenol) were observed, corroborating the results found in this work [32].

### 2.3. Antimicrobial Evaluation

The results obtained for Minimal Inhibitory Concentration (MIC) of the EO studied are shown in Table 2. The EO exhibited strong to moderate antimicrobial activity, according to Vuuren and Holl [33], against five of the tested microorganisms, with MIC values ranging from 0.062 to 1.0 mg/mL. The highest activities (0.062 mg/mL) were observed for *B. uncinella* EO from spring against *S. aureus*, *C. albicans* and *B. uncinella* EO from summer against *C. albicans* and *B. subtilis*. However, no antimicrobial activity was observed against *P. aeruginosa* and *S. epidermidis* up to an EO concentration of 2.0 mg/mL.

Cazella [35] evaluated *B. dracunculifolia* EO against eight pathogenic agents and reported bacteriostatic and bactericidal activities, mainly against *S. aureus*, *B. cereus* and *P. aeruginosa*, as well as fungistatic and fungicidal activities. These authors explained that antibacterial activity was more effective than the antifungal one by using the essential oil at lower concentrations pointing to its use as a potential alternative for food applications to reduce synthetic chemicals in a more sustainable food industry.

Although the EO in our study did not show antimicrobial activity against *P. aeruginosa*, in contrast to the findings by Cazella [35], our results demonstrated antimicrobial effects at lower EO concentrations against *S. aureus* and *E. coli*, also studied by the authors.

Freitas [36] evaluated *B. coridifolia* EO composition, which showed germacrene D, bicyclogermacrene and E-caryophyllene as major components. The antimicrobial tests showed effects on the control of *P. aeruginosa* and *S. aureus*. Antimicrobial evaluation of *B. erioclada* EO revealed moderate activity (1000 μg/mL in both *E. coli* and *C. albicans* and >2000 μg/mL in *P. aeruginosa* and *S. aureus* [37].

In our study, for *B. uncinella*, the spring EO had high levels of caryophyllene oxide, and the summer EO had high levels of *α*-pinene. Both EO had high levels of limonene and spathulenol. These compounds have been described as having potential antimicrobial activity.

An interesting aspect to consider is the potential synergistic effects among the different compounds present in the essential oils. The antimicrobial activity observed from *Baccharis* species essential oils may be attributed not only to the isolated action of the major compounds but also to their interactions. According to Bakkali [38], essential oils, composed of a complex mixture of terpenes, sesquiterpenes, and other compounds, can display enhanced antimicrobial effects due to their combined action. This phenomenon arises because individual compounds in the EO may target different cellular mechanisms, leading to a more potent overall antimicrobial effect compared to the action of individual compounds in isolation.

The antibacterial effects of *α*- and *β*-pinene were described due to their toxic effects on membranes [39]. Borges [40], in a review study on *α*-pinene and antimicrobial activity, found that this compound has antibacterial properties in isolated form (40% of studies) or in synergistic application. Its effect was shown in strains of *E. coli*, *S. aureus* and *S. enterica*, and current bacterial strains revealed its susceptibility.

The antibacterial properties of limonene are well known and discussed in several studies against many bacterial species [41]. Han [42] studied the antimicrobial activity and mechanisms of action of limonene and observed that this compound has a potent inhibitory activity against *S. aureus*, destroying the cell morphology and integrity of the cell wall of the bacteria.

Spathulenol was related in higher amounts in summer for another *Baccharis* species [6,20]. A study from wild species of *B. dracunculifolia* Tomazzoli [29] found a negative correlation between this compound and antioxidant activity, suggesting its presence as an oxidant inhibitor in higher temperatures.

Santana [43] observed that *trans*-caryophyllene and caryophyllene oxide, when used individually, possibly act as inhibitors of the efflux pumps NorA, Tet(K), MsrA and MepA in resistant strains of *S. aureus*. Furthermore, this suggests a potential increase in the action exerted by the antibiotic, observed by the reduction in the MIC of the antibiotic after association with the substances tested.

In another study, where caryophyllene oxide is the major compound in the EO of *Myrcia oblongata*, it was observed that it presents an inhibitory activity for the Gram-positive bacteria, *S. aureus* and *B. subtilis* and for the yeast *C. albicans* [43].

In the study of the EO from the leaves of *Eugenia pyriformis*, where spathulenol is also the major compound, antimicrobial activity was observed against *S. aureus*, *E. coli* and *P. aeruginosa* [44].

The results in Table 2 also showed antimicrobial activity only at higher temperatures (spring and summer), which may suggest a synergistic action between EO compounds during these seasons.

Another biological analysis, as performed by Budel [45], of five *Baccharis* species revealed potential results in insecticidal activities (*B. pauciflosculosa*, *B. reticularioides* and *B. sphenophylla*) and exhibited moderate antimalarial activities (*B. microdonta* and *B. punctulata*).

While *Thymus vulgaris* essential oil (thymol chemotype) is well known and commonly used for its potent antimicrobial properties, the results from a previous study conducted by our research team at CPQBA-State University of Campinas—Brazil [34] showed that *Thymus* vulgaris essential oil (containing 79.15% of thymol) exhibited lower antimicrobial activity compared to *Baccharis* essential oil. In this study, *Baccharis* essential oil demonstrated higher antimicrobial activity for the same microorganisms tested, while *Thymus* essential oil showed lower activity, suggesting that *Baccharis* essential oil may have greater potential for practical applications in antimicrobial treatments. It is important to highlight that the antimicrobial activity data referenced for *Thymus vulgaris* essential oil were obtained from a previous study and not under the same experimental conditions as those applied to *Baccharis* in this study. There is inherent variability in antimicrobial susceptibility results due to factors such as seasonality and regionality, all of which can directly influence the chemical composition of essential oils and their observed bioactivity.

As there is no consensus regarding the acceptable inhibition levels for natural products compared to standard antibiotics, we adopted the classification proposed by Vuuren and Holl [33], which defines strong activity as MIC ≤ 0.5 mg/mL, moderate activity as MIC between 0.51 and 1.0 mg/mL, and weak activity as MIC > 1.0 mg/mL. Accordingly, the results obtained in the present study demonstrate a broad-spectrum action of *Baccharis* essential oils, with MIC values ranging from strong to moderate activity.

### 2.4. Anti-Proliferative Activity Evaluation

The anti-proliferative activity was expressed as the concentration required to induce total cell growth inhibition (TGI) [46]. Considering the mean TGI values against tumor cell lines and the CSIR’s criteria [inactive (I, TGI ≥ 50 µg/mL), weak (W, 15 µg/mL ≤ TGI < 50 µg/mL), moderate (M, 6.25 µg/mL ≤ TGI < 15 µg/mL) or potent (P, TGI < 6.25 µg/mL) activity] to classify anti-proliferative activity [47].

The cytostatic effect of the seasonal samples of *Baccharis* EO was evaluated following the NCI protocol for anti-proliferative activity. In this protocol, untreated cells were evaluated before (T0) and after (T1) the exposure time, allowing to distinguish cytostatic and cytocidal effects. The exposure time (48 h) was selected to allow at least one complete cell cycle for each cell line [46]. As recommended by Monks [46], doxorubicin was used as a positive control in a concentration range (0.025–25 µg/mL) lower than that recommended for crude extracts (0.25–250 µg/mL). The results described in Table 3 for doxorubicin reflect the variability in the sensitivity of tumors from different tissues to a chemotherapeutic agent.

*Baccharis calvescens* EO showed more effective anti-proliferative activity (mean TGI ranging from <7.4 to 42 µg/mL) in comparison to *B. retusa* EO (mean TGI ranging from 25 to 53 µg/mL) and *B. uncinella* EO (mean TGI ranging from 38 to >106 µg/mL) (Table 3).

For *B. calvescens* EO, the harvest during winter (August, mean TGI = 11 µg/mL) and spring (November, mean TGI < 7 µg/mL) resulted in moderate cytostatic effects, while EO obtained from aerial parts collected in summer (January, mean TGI = 42 µg/mL) and autumn (May, mean TGI = 18 µg/mL) showed weak anti-proliferative activity (Table 3). Considering the most active sample as *Baccharis calvescens* EO obtained from aerial parts collected in spring, the most sensitive cell lines to EO were MCF-7 (breast, adenocarcinoma; TGI < 0.25 µg/mL) and OVCAR-03 (ovary, adenocarcinoma; TGI < 0.25 µg/mL) cell lines (Table 3).

The *B. retusa* EO aerial parts collected in autumn were inactive EO (mean TGI = 53 µg/mL), while the other three samples corresponding to collects during winter, spring and summer showed weak cytostatic effects (mean TGI ranging from 25 to 46 µg/mL). Interestingly, despite showing a mean weak effect, the summer sample of *B. retusa* EO showed a potent cytostatic effect against the K562 cell line (chronic myelogenous leukemia; TGI = 2 µg/mL) and moderate activity against UACC-62 (melanoma; TGI = 9 µg/mL). Moreover, the spring sample of *B. retusa* EO potently inhibited the cell growth of the OVCAR-03 (ovary, adenocarcinoma; TGI = 4 µg/mL) cell line (Table 3).

Finally, for *B. uncinella* EO aerial parts collected in autumn and winter provided an inactive EO (mean TGI = 57 and >106 µg/mL, respectively), while the samples corresponding to collects during spring and summer showed weak cytostatic effects (mean TGI = 38 µg/mL). Spring sample of *B. uncinella* EO showed potent anti-proliferative activity against the K562 cell line (chronic myelogenous leukemia; TGI = 3 µg/mL) and moderate cytostatic effect against OVCAR-03 (ovary, adenocarcinoma; TGI = 10 µg/mL) cell line (Table 3).

Considering the chemical composition, both the *B. calvescens* spring EO and *B. uncinela* the spring EO showed high levels of spathulenol. For *B. retusa*, the summer and spring EOs present high levels of limonene and α-pinene, respectively. According to the literature, several essential oils containing spathulenol and/or α-pinene as the main compound show promising anti-proliferative effects against different types of cancer, such as breast, colon and ovary [44,48]. Observations in cells, animal and epidemiological models showed that limonene altered cancer cell signaling pathways, preventing their proliferation and causing apoptosis. In animal models, limonene slowed the growth of cancer of the pancreas, stomach, colon, skin and liver [49].

## 3. Materials and Methods

### 3.1. Plant Material

Leaves of *B. uncinella*, *B. retusa* and *B. calvescens* were collected in Piraquara—Paraná (25°30′26″ S, 49°01′36″ W)—Southern Hemisphere (morning period). For each sample, plant material was collected in the middle of each season [Winter (June–September), Spring (September–December), Summer (December–March) and Autumn (March–June)] of the year from a group of individuals (collection radius of approximately 1500 m^2^). The samples were identified by Dr. Gustavo Heiden (Embrapa Clima Temperado–RS). The voucher specimens (*B. uncinella*—HMBM 9119; *B. calvescens*—HMBM 9116; *B. retusa*—HMBM 9118) were deposited in the Herbarium of the Municipal Botanical Museum of Curitiba.

### 3.2. Isolation and Analysis of Essential Oil

Leaves were dried in a circulating air oven at 40 °C until constant mass. Dried aerial parts of each plant were submitted to hydrodistillation in a modified Clevenger apparatus for 4 h (70 g/1000 mL of distilled water). All extractions were performed in triplicate. After the extraction, the essential oil was collected and stored under refrigeration until the analysis by GC-MS. The yield was calculated by relating to the volume of EO obtained and the mass of dry material used in the extraction (% v/m).

The GC-MS analyses were carried out on a Shimadzu^®^ (Kioto, Japan) GCMS-QP2010 Plus gas chromatograph coupled to a mass selective detector, using fused silica capillary column Rtx-5MS (30 m × 0.25 mm × 0.25 µm). Helium was used as carrier gas (57.4 kPa, 1.0 mL/min). The column temperature program was 60 °C to 250 °C with a heating rate of 3 °C/min. The injector temperature was adjusted at 250° C. The injected sample volume was 1.0 μL, in split mode (1:10). The mass spectra were recorded at 70 eV. The Arithmetical retention indices (AI) were calculated using Vanden Dool and Kratz’s [24] equation using a mixture of *n*-alkanes (C10–C24) analyzed in the same chromatographic conditions. The substance identification was attained by comparison of experimental data (calculated AI and mass spectrum of each chromatographic peak) with those described in the literature [50] and the NIST 08 library of fragmentation patterns.

The quantification was determined by GC-FID analyses using Agilent 7890A equipment (Santa Clara, CA, USA) with a flame ionization detector (280 °C) and a Shimadzu SH-Rtx-5MS fused silica capillary column (30 m × 0.25 mm × 0.25 μm).

#### Statistical Analysis

The chemical variation in the constituents of the EO was evaluated through Principal Components Analysis (PCA) performed with PLS-Toolbox 3.0 (Eigenvector Research, Inc., Manson, WA, USA) software operated in Matlab 7.0.1 (MathWorks^®^). PCA was used to extract relevant information from the data and to provide a simultaneous representation of the samples (scores), considering the chemical composition of the essential oils (loadings), to generate groupings or separations between them. The pre-processing of data used in PCA was autoscaling.

The data matrices were generated from the identification and relative quantification of the EO. For the simultaneous analysis of the three species, a matrix with 138 rows and 12 columns was generated (See Table 1). For *B. uncinella* EO, the data matrix was 77 rows and 4 columns; for *B. retusa*, there is 87 rows and 4 columns; for *B. calvescens*, there is 84 rows and 4 columns. The rows correspond to the identified compounds (their respective concentration being assigned to each sample), and the columns correspond to the extracted oil samples from the different seasons of the year.

The average concentrations of the triplicates were considered for all analyses. All graphs were generated using Origin 8.5 software.

### 3.3. Biological Activity Screening

#### 3.3.1. Antimicrobial Activity

The minimal inhibitory concentration (MIC) of the EO from the leaves of *B. uncinella* collected in the four seasons, as well as those from *B. retusa* and *B. calvescens* collected in spring, summer, and autumn, were tested against *Candida albicans* (ATCC 10231), *Bacillus subtilis* (ATCC 6051), *Pseudomonas aeruginosa* (ATCC 13388), *Staphylococcus aureus* (ATCC 6538), *Escherichia coli* (ATCC 11775), *Salmonella choleraesuis* (ATCC 10708) and *Staphylococcus epidermidis* (ATCC 12228) according to the guidelines of the Clinical and Laboratory Standards Institute [51,52].

For the preparation of the inoculum, the microorganisms were grown overnight at 36 °C in Nutrient Agar (NA Difco^TM^, Detroit, MI, USA) for bacteria and Sabouraud Dextrose Agar (SDA Merck, Darmstadt, Alemanha) for yeast. The cell mass was diluted into a solution of NaCl (0.85%), the turbidity was adjusted to 0.5 on the McFarland scale and confirmed in a spectrophotometer (Shimadzu UV mini 1240 Spectrophotometer) at 530 nm (*C. albicans*) or 625 nm (bacteria) to absorbance between 0.08 and 0.1 (10^6^ cell/mL for yeast and 10^8^ cell/mL for bacteria). The cell suspensions were finally diluted to 10^4^ cells/mL (yeast) or 10^6^ cells/mL (bacteria).

For the MIC analysis, 96-well tissue culture microplates containing 100 µL of Muller-Hinton Broth (NA Difco^TM^) (bacteria) and RPMI-1640 (Sigma^®^, St. Louis, MO, USA) (yeast) were used. The stock solutions of the EO were diluted to 8 mg/mL and transferred to the first well, and serial dilutions were performed to reach concentrations ranging from 2.0 to 0.098 mg mL^−1^. Nystatin (Sigma^®^) and chloramphenicol (Sigma^®^) were used as antimicrobial standards. The inoculum (100 µL) was added to all the wells, and the microplates were incubated for 24 h (bacteria) and 48 h (yeast). The inhibition of microorganisms was confirmed by the addition of 50 µL of a triphenyl tetrazolium solution (0.1%) (Sigma^®^) for the bacteria. After one hour at 37 °C, the last well without red coloration was visually identified as MIC. For yeast, a change in the color of the nutrient media indicated growth. The lowest concentration of the EO that visibly inhibited microbial growth was defined as the MIC. The tests were performed in triplicate.

#### 3.3.2. Anti-Proliferative Activity Assay

The anti-proliferative activity evaluation was performed against a panel of nine human tumor cell lines [U251 (glioblastoma); UACC-62 (melanoma); MCF-7 (breast adenocarcinoma); NCI-ADR/Res (ovarian multidrug-resistant adenocarcinoma); 786-0 (kidney, adenocarcinoma); NCI-H460 (lung, non-small cell carcinoma); OVCAR-03 (ovarian, adenocarcinoma); HT-29 (colorectal adenocarcinoma); K562 (chronic myelogenous leukemia)] kindly provided by the Frederick Cancer Research & Development Center, National Cancer Institute, MA, USA. One human non-tumor cell line (HaCaT, immortalized keratinocytes) was acquired from the Rio de Janeiro Cell Bank (BCRJ, Br).

All human cell lines were cultured in complete medium [RPMI-1640 (Gibco, Tulsa, OK, USA) supplemented with 5% Fetal Bovine Serum (Gibco, cat. n. A5670701) and 1% of penicillin/streptomycin solution (1000 U mL^−1^:1000 mg mL^−1^) (Vitrocell^®^, Campinas, SP, Brazil)], incubated at 37 °C and 5% CO_2_ and subcultured each 4–5 days using trypsin/EDTA solution. The experiments were carried out with cells in passages 5 to 12.

Aliquots (5.0 mg) of each EO from *Baccharis* species were solubilized in DMSO (50 μL) using ultrasound, followed by dilution in complete medium (950 μL), obtaining a homogeneous dispersion which was immediately subjected to serial dilution in complete medium, obtaining the final concentrations of 0.25, 2.5, 25, and 250 μg·mL^−1^. Doxorubicin (Eurofarma, SP, Brazil) was diluted in the same way, obtaining the final concentrations of 0.025, 0.25, 2.5 and 25 μg·mL^−1^. As previously reported [53,54,55], the final concentration of DMSO (≤0.15%) did not affect cell viability.

The cells were seeded in 96-well plates (100 μL/well, cell density 3 to 6·10^3^ cells/well) and left to recover for 24 h before samples were added in triplicate (100 μL/well; 0.25 to 250 μg·mL^−1^) and incubated for 48 h at 37 °C and 5% CO_2_. Doxorubicin was used as a positive control (0.025 to 25 μg·mL^−1^, in triplicate). The cells were fixed with trichloroacetic acid (50%, 50 μL/well) before (T0) and after (T1) the addition of the sample. Cell content was quantified using the sulforhodamine B protocol (SRB 0.4% in 1% aqueous acetic acid solution) at 540 nm. The proliferation (%) of each cell line in the presence of each sample concentration was calculated based on the difference between the T1 and T0 absorbance values of untreated cells as representing 100% cell proliferation. Results were expressed as cell growth versus sample concentration curves, and the sample concentration required for total cell growth inhibition (TGI) of each cell line was calculated by sigmoidal regression using Origin 8.0 software [46,54,56].

## 4. Conclusions

The study of the chemical composition of the EO of *Baccharis calvescens*, *B. retusa* and *B. uncinella* showed differences between the species. For each species, the chemometric analysis showed that seasonal variation influenced the quantitative and qualitative chemical composition of the EO. Understanding the impact of seasonality on chemical composition can help improve compound production to better meet the increasing demand for phytochemicals in the context of a rapidly changing climate.

The EO of *B. calvescens* showed more effective anti-proliferative activity in the winter and spring seasons. Regarding the antimicrobial analysis, the greatest activities were observed for the EO of *B. uncinella* from spring and summer. It is important to highlight that these oils have spathulenol and caryophyllene oxide as their major compounds, except for *B. uncinella* in summer, where spathulenol and α-pinene stand out. This suggests that these compounds may be primarily responsible for the activity observed in these species.

This is the first study of *B. retusa* EO. The major compounds were limonene (summer), germacrene-D (autumn), α-pinene (spring) and spathulenol (winter). Antimicrobial and antitumor analyses showed promising results, with the summer *B. retusa* EO sample showing a potent cytostatic effect against the K562 cell line. Furthermore, the spring *B. retusa* EO sample potently inhibited cell growth of the OVCAR-03 cell line. This encourages continued studies to better understand the anti-proliferative activity of these oils.

This study offers valuable insights into the seasonal variation in the chemical composition of EO from *B. calvescens*, *B. retusa* and *B. uncinella* and how this variation is linked to their biological activity. Key compounds such as spathulenol, caryophyllene oxide, limonene and α-pinene appear to play a significant role in the observed antimicrobial and anti-proliferative effects. As demonstrated, the concentration of these compounds varies across different seasons and species. By better aligning biological activity with the collection season, it is possible to recommend summer and spring for *B. uncinella* and *B. retusa* and winter and spring for *B. calvescens*, with the goal of optimizing the extraction of bioactive phytochemicals to meet the increasing demand in a rapidly changing climate.

## Figures and Tables

**Table 1:** The chemical composition of essential oils based on the seasonality of leaves of *Baccharis calvescens*, *B. retusa* and *B. uncinella*.

Nº	AI	Compound	Relative Content (%) ^b^											
			*Baccharis calvescens*				*Baccharis retusa*				*Baccharis uncinella*			
			Winter	Spring	Summer	Autumn	Winter	Spring	Summer	Autumn	Winter	Spring	Summer	Autumn
1	920	α-Thujene	0.3 ± 0.1	0.7 ± 0.2	1.1 ± 0.1	0.8 ± 0.3	5 ± 3	8.7 ± 1.6	5.0 ± 1.6	4.1 ± 1.2	0.8 ± 0.8	1.9 ± 0.3	2.4 ± 0.2	2.2 ± 0.5
2	928	α-Pinene	2 ± 1	2.2 ± 0.4	4.2 ± 0.4	2.6 ± 0.5	6 ± 3	11 ± 3.0	5.5 ± 1.5	5.2 ± 1.4	2.5 ± 1.9	4.7 ± 0.9	6.1 ± 0.4	5.8 ± 1.0
3	939	α-Fenchene	0	0	0	0	0	0	0.4 ± 0.2	0	0	0	0	0
4	966	Sabinene	0.2 ± 0.2	0.4 ± 0.2	0.3 ± 0.3	0.6 ± 0.2	1.9 ± 1.1	2.0 ± 1.3	4.0 ± 0.2	0.8 ± 0.2	0.6 ± 0.3	0.8 ± 0.2	1.2 ± 0.3	1.4 ± 0.4
5	971	β-Pinene	2 ± 1	3.2 ± 0.7	4.7 ± 0.6	4.4 ± 0.9	3.4 ± 1.6	5.9 ± 0.5	5.6 ± 0.1	2.7 ± 0.6	2 ± 1	3.6 ± 0.6	3.5 ± 0.2	3.8 ± 0.7
6	984	Myrcene	0	0	0.5 ± 0.1	0.9 ± 0.2	2.0 ± 0.7	2.4 ± 0.2	2.1 ± 0.1	1.7 ± 0.4	0.6 ± 0.2	0.8 ± 0.1	1.10 ± 0.03	0
7	999	α-Phellandrene	0	0	0	0	0.3 ± 0.3	0.7 ± 0.2	0.75 ± 0.04	0.7 ± 0.2	0	0	0	0
8	1003	δ-3-Carene	0	0	0	0	0.4 ± 0.3	0	0	0	0	0	0	0
9	1012	α-Terpinene	0	0	0.3 ± 0.3	0.2 ± 0.1	0.5 ± 0.2	1.3 ± 0.2	1.37 ± 0.03	0.8 ± 0.3	0.3 ± 0.1	0.6 ± 0.1	1.1 ± 0.1	0.5 ± 0.1
10	1016	*p*-Cymene	0	0	0.4 ± 0.2	0	1.6 ± 0.4	1.3 ± 0.4	1.3 ± 0.6	0.6 ± 0.1	0.23 ± 0.03	0.18 ± 0.01	0.8 ± 0.1	0.7 ± 0.2
11	1022	*o*-Cymene	0	0	0	0	0	0	0	0.6 ± 0.1	0	0	0	0
12	1025	Limonene	1.0 ± 0.4	2.9 ± 0.5	3.2 ± 0.4	2.4 ± 0.4	2.5 ± 0.7	6.4 ± 0.3	12 ± 0.0	3.7 ± 0.0	3.9 ± 0.9	5.3 ± 0.8	5.8 ± 0.4	4.8 ± 0.44
13	1036	(*Z*)-β-Ocimene	0	0	0	0	0	0.5 ± 0.2	0.6 ± 0.1	0	0	0	0	0
14	1044	(*E*)-β-Ocimene	0	0	0	0.9 ± 0.2	0.8 ± 0.3	0	0	0.5 ± 0.1	0	0	0	0
15	1052	γ-Terpinene	0.1 ± 0.1	0	0.5 ± 0.1	0.3 ± 0.1	1.0 ± 0.2	2.1 ± 0.5	1.6 ± 0.1	1.0 ± 0.9	0.6 ± 0.1	0.9 ± 0.2	1.4 ± 0.1	0.7 ± 0.1
16	1058	*cis*-Sabinene hydrate	0	0	0	0	0.3 ± 0.1	0	0.3 ± 0.1	0.4 ± 0.6	0	0	0	0
17	1078	Mentha-2,4(8)-diene	0	0	0	0	0	0.8 ± 0.1	0.9 ± 0.1	0	0	0	0	0
18	1082	Terpinolene	0	0	0	0	0.5 ± 0.1	0	0	0.6 ± 0.2	0.3 ± 0.3	0.2 ± 0.1	0.9 ± 0.1	0.24 ± 0.02
19	1086	*p*-Cymenene	0	0	0	0	0	0.20 ± 0.03	0	0.3 ± 0.1	0	0	0	0
20	1091	Linalool	0.1 ± 0.2	0	0.7 ± 0.1	0	0	0.3 ± 0.03	0	0.3 ± 0.1	0	0	0.64 ± 0.04	0
21	1092	*trans*-Sabinene hydrate	0	0	0	0	0	0	0.4 ± 0.1	0	0	0	0	0
22	1106	Perillene	0	0	0	0	0	0.2 ± 0.1	0.5 ± 0.04	0	0	0	0	0
23	1107	*trans*-Thujone	0	0	0	0	0	0	0.3 ± 0.3	0	0	0	0	0
24	1112	*cis*-*p*-Menth-2-en-1-ol	0	0	0	0	0	0	0.8 ± 0.04	0	0	0	0.53 ± 0.04	0
25	1115	*trans*-*p*-Mentha-2,8-dien-1-ol	0	0	0	0	1 ± 0.4	0	0	0	0	0	0	0
26	1118	α-Campholenal	0	0	0	0	0.7 ± 0.1	0	0	0	0	0	0	0
27	1135	*trans*-Pinocarveol	0.1 ± 0.2	0	1.7 ± 0.4	0	0.9 ± 0.1	0.3 ± 0.3	0	0	0	0	0.8 ± 0.1	0.33 ± 0.03
28	1136	*cis*-Verbenol	0	0	0	0	0.5 ± 0.1	0.2 ± 0.1	0	0	0	0	0	0
29	1138	*trans*-Verbenol	0	0	1.1 ± 0.1	0	1.7 ± 0.4	0	0.5 ± 0.2	0	0	0	0.3 ± 0.3	0
30	1158	Isoborneol	0	0	0	0	0	0.3 ± 0.3	0	0	0	0	0	0
31	1160	Pinocarvone	0	0	1.3 ± 0.3	0	0.3 ± 0.1	0	0.4 ± 0.04	0	0	0	0.41 ± 0.04	0
32	1167	Borneol	0	0	0	0	0	0	0	0.4 ± 0.1	0	0	0	0
33	1168	*p*-Mentha-1,5-dien-8-ol	0	0	0.7 ± 0.3	0	0.8 ± 0.2	0	0.7 ± 0.2	0	0	0	0.7 ± 0.2	0.16 ± 0.01
34	1173	Terpinen-4-ol	0.1 ± 0.2	0.6 ± 0.1	1.2 ± 0.3	0.4 ± 0.3	2.0 ± 0.4	2.8 ± 0.2	2.9 ± 0.3	2.4 ± 0.5	1.3 ± 0.3	1.5 ± 0.3	2.9 ± 0.3	1.4 ± 0.2
35	1175	Naphthalene	0	0	0	0	1.7 ± 0.3	0	0	0	0	0	0	0
36	1179	Cryptone	0	0	0	0	0	0	1.7 ± 0.2	0	0	0	0	0
37	1186	α-Terpineol	0	0	0.9 ± 0.2	0.2 ± 0.2	0.5 ± 0.2	1.3 ± 0.1	1.2 ± 0.02	0.7 ± 0.6	0.4 ± 0.1	0.5 ± 0.1	1.3 ± 0.2	0.5 ± 0.1
38	1190	Myrtenol	0	0	2.2 ± 0.5	0	0.9 ± 0.2	0	0.8 ± 0.2	0	0	0	0.7 ± 0.1	0.3 ± 0.1
39	1210	*trans*-Carveol	0	0	0.7 ± 0.2	0	0	0	0.2 ± 0.2	0	0	0	0.5 ± 0.1	0
40	1230	Cumin aldehyde	0	0	0	0	0	0	0.7 ± 0.1	0	0	0	0	0
41	1234	Carvone	0	0	0.4 ± 0.4	0	0	0	0	0	0	0	0	0
42	1264	*p*-Menth-1-en-7-al	0	0	0	0	0	0	1.4 ± 0.3	0	0	0	0	0
43	1274	α-Terpinen-7-al	0	0	0	0	0	0	0.4 ± 0.1	0	0	0	0	0
44	1330	δ-Elemene	0	0	0	0	0.7 ± 0.1	0.8 ± 0.4	0.5 ± 0.1	0.7 ± 0.1	0	0.2 ± 0.1	0.5 ± 0.1	0.25 ± 0.02
45	1371	α-Copaene	0.9 ± 0.4	1.0 ± 0.1	0.4 ± 0.1	0.6 ± 0.1	0	0	0.5 ± 0.1	1.0 ± 0.1	0.4 ± 0.2	0.5 ± 0.2	0	0.25 ± 0.02
46	1386	β-Bourbonene	0	2.3 ± 0.1	0	0.61 ± 0.03	0	0	0	0	0	0	0	0
47	1390	β-Elemene	0.2 ± 0.4	0	1.0 ± 0.1	4.2 ± 0.8	1.0 ± 0.2	1.1 ± 0.6	1.1 ± 0.1	1.8 ± 0.1	0.3 ± 0.1	0.3 ± 0.1	0.1 ± 0.2	1.07 ± 0.04
48	1409	(*Z*)-Caryophyllene	0	0	2.5 ± 0.3	0	5.0 ± 1.4	5.0 ± 0.6	4.4 ± 0.1	0	2.9 ± 0.4	4.3 ± 0.2	2.3 ± 0.4	0
49	1412	α-Gurjunene	0	0	0	0	0	0	0	0	0	0.2 ± 0.1	0.9 ± 0.1	0.31 ± 0.03
50	1423	(*E*)-Caryophyllene	4 ± 2	4.7 ± 0.3	0	5.96 ± 0.06	0	0	0	9.1 ± 0.2	0	0	0	4.6 ± 0.2
51	1435	γ-Elemene	0	0	0	0	0	0.4 ± 0.2	0.3 ± 0.1	0.3 ± 0.3	0	0	0	0
52	1436	α-Guaiene	0	0	0	0	0	0.8 ± 0.1	0	0	0	0	0	0
53	1444	Aromadendrene	0	0	0	0	0	0	0.9 ± 0.2	0.8 ± 0.1	0	0	0	0
54	1451	α-Humulene	0.8 ± 0.7	2.9 ± 0.3	1.8 ± 0.2	4.6 ± 0.3	0.8 ± 0.0	0.5 ± 0.2	0	1.2 ± 0.1	1.05 ± 0.04	1.3 ± 0.2	0.9 ± 0.1	1.4 ± 0.1
55	1460	*allo*-Aromadendrene	0.2 ± 0.3	0.4 ± 0.1	0	0	0	0	0	0	0	0	0	0
56	1462	*cis*-Cadina-1(6),4-diene	0	0	0	0	0	0.6 ± 0.1	0	0	0.38 ± 0.04	0.5 ± 0.1	0	0
57	1464	9-*epi*-(*E*)-Caryophyllene	0	0	0	0	0	0	0	1.0 ± 0.1	0	0.5 ± 0.1	0	0.22 ± 0.02
58	1465	*cis*-Muurola-4(14),5-diene	0	0	0.6 ± 0.1	0	0	0	3.3 ± 0.2	0	0	1.4 ± 0.3	0	0
59	1471	Dauca-5,8-diene	0.2 ± 0.4	0.31 ± 0.02	0.8 ± 0.3	0.2 ± 0.2	0	5 ± 2	0	0.1 ± 0.1	0	0	0	0
60	1477	*trans*-Cadina-1(6),4-diene	0	0	0	1.2 ± 0.1	0	0	0	0	0	0	0	0
61	1478	γ-Gurjunene	0	0	0	0	0	0	0	0	0	0.6 ± 0.4	0	0
62	1479	γ-Muurolene	0.6 ± 0.5	0.6 ± 0.1	0.7 ± 0.6	1.8 ± 0.3	5.4 ± 2.1	0	0	0	1.3 ± 0.4	0.1 ± 0.1	0.6 ± 0.1	0
63	1481	γ-Himachalene	0	0	0.2 ± 0.4	0	0	0	0	0	0	0	0	0
64	1483	γ-Curcumene	0	0	0	0	0	6.1 ± 0.8	0	0	0	0	0	0
65	1485	α-Amorphene	0	0	0.4 ± 0.3	0	0	0.7 ± 0.6	0	0	0	0.5 ± 0.1	0	0
66	1486	Germacrene D	5 ± 1	1.6 ± 0.1	1.4 ± 0.2	6.6 ± 0.3	0	0	0	11.7 ± 0.7	0	0	0	0
67	1487	*cis*-Eudesma-6,11-diene	0.6 ± 0.1	0	0	0	0	0	0	0	0	0	0	0
68	1489	β-Selinene	0.2 ± 0.1	0.9 ± 0.2	0	1.63 ± 0.02	0	0	0	0	0	0	0	0
69	1492	*trans*-Muurola-4(14),5-diene	0.2 ± 0.3	0.4 ± 0.1	0	1.05 ± 0.04	0	0	0	0	0	0	0	0
70	1497	γ-Amorphene	0.7 ± 0.6	0	0	0	0	0	0	0	0	0	0	0
71	1499	Viridiflorene	0	1.6 ± 0.1	0	1 ± 2	0	0	0	0	0.6 ± 0.2	0	0	0
72	1500	Bicyclogermacrene	2.7 ± 0.9	1.4 ± 0.4	0	5 ± 2	5 ± 1	0	2.8 ± 0.4	8.3 ± 0.1	1.1 ± 0.1	0	1.4 ± 0.3	0
73	1501	α-Muurolene	0.7 ± 0.1	0.6 ± 0.1	0	0.3 ± 0.6	0	0	0	0	0.6 ± 0.2	0.4 ± 0.1	0.5 ± 0.1	2.78 ± 0.04
74	1510	Germacrene A	0	0	0	1.18 ± 0.09	0	0	0	0	0	0	0	0
75	1511	δ-Amorphene	0	0	1.8 ± 0.3	0	1.0 ± 0.1	0	1.2 ± 0.1	0.6 ± 0.0	0	1.8 ± 0.2	1.3 ± 0.1	0
76	1512	*trans*-Cycloisolongifol-5-ol	0	0	0	0	0	0	0	0	0.7 ± 0.3	0	0	0
77	1514	γ-Cadinene	0.1 ± 0.2	0.3 ± 0.3	0	0.8 ± 0.1	0	0	0	1.0 ± 0.1	0.5 ± 0.1	0.1 ± 0.2	0	0
78	1518	Cubebol	0.3 ± 0.6	0.06 ± 0.04	0	0.9 ± 0.1	0	0	0	0	0	0	0	0
79	1519	(*Z*)-γ-Bisabolene	1 ± 1	0	0	0	0	0	0	0	0	0	0	0
80	1521	δ-Cadinene	2 ± 1	2.5 ± 0.2	0	3.9 ± 0.1	0	1.4 ± 0.1	0	2.4 ± 0.2	2.0 ± 0.5	0	0	0.74 ± 0.05
81	1537	*trans*-Cadina-1,4-diene	0	0	0	0.3 ± 0.3	0	0	0	0	0	0	0	0
82	1542	α-Cadinene	0	0	0	0	0	0	0	0.30 ± 0.0	0	0	0	0
83	1548	α-Calacorene	0.3 ± 0.3	0.7 ± 0.1	0.7 ± 0.1	0.5 ± 0.1	0	0	0	0.30 ± 0.0	0	0	0	0
84	1551	Elemol	0	0	0	1.2 ± 0.2	0	0	0	0	0.08 ± 0.14	0	0	1.29 ± 0.09
85	1554	Germacrene B	0	0	0	0	0	0.9 ± 0.5	0.9 ± 0.0	1.3 ± 0.4	0	0	0	0
86	1560	(*E*)-Nerolidol	0	0	1.3 ± 0.1	1 ± 0	0.40 ± 0.4	0.5 ± 0.1	0.4 ± 0.1	0.8 ± 0.1	0.5 ± 0.2	0.8 ± 0.1	0.9 ± 0.8	0.4 ± 0.0
87	1565	β-Calacorene	0	0.4 ± 0.1	0	0	0	0	0	0	0	0	0	0
88	1566	Maaliol	0	0	0	0	0	0.5 ± 0.1	0.5 ± 0.1	0.7 ± 0.1	0.2 ± 0.3	0.9 ± 0.1	0	0
89	1567	Palustrol	0	0	0	0.6 ± 0.0	0.3 ± 0.3	0	0	0	0	0	0	0
90	1572	Longipinanol	0	0	0	0	0	0	0	0	0	0	0	0.8 ± 0.1
91	1577	Spathulenol	15 ± 3.0	10 ± 1.0	13 ± 1.0	2.9 ± 0.2	10 ± 3	9 ± 3	8.1 ± 0.4	4.3 ± 0.5	11 ± 1	11.0 ± 0.6	15 ± 1	14.6 ± 0.8
92	1578	Caryophyllene oxide	12 ± 2.0	7 ± 1	5 ± 1	3.2 ± 0.2	7 ± 4	4 ± 1	2.5 ± 0.5	0	5 ± 2	6.6 ± 1.8	0	0
93	1582	Thujopsan-2α-ol	0	0	0	0	0	0	0	0	0	1.29 ± 0.04	0	0
94	1585	*allo*-Hedycaryol	0	0	0	0.7 ± 0.1	0	0	0	0	0	0	0	0
95	1590	Globulol	1.1 ± 0.1	0	1.2 ± 0.04	0	1.0 ± 0.2	1.2 ± 0.2	0	3.9 ± 0.5	0	2.8 ± 0.3	1.9 ± 0.3	5.6 ± 0.4
96	1593	Viridiflorol	0	0.3 ± 0.6	0	1.04 ± 0.06	0	0	0	1.8 ± 0.3	0	0	0	0
97	1594	Salvial-4(14)-en-1-one	0	0	0	0	0	0	0	0	2.2 ± 0.2	0	0	0
98	1597	*cis*-dihydro-Mayurone	0	0	0	0	0	0	0	0	0	1.5 ± 0.4	0	0
99	1604	Rosifoliol	0	0	0	0	0	0	0	1.1 ± 0.2	0	0	0	0
100	1605	Ledol	1.9 ± 0.8	0	0.99 ± 0.02	1.2 ± 0.3	0	0	0	0	0	0	0	0
101	1607	Humulene epoxide II	2.2 ± 0.3	4 ± 1	5.7 ± 0.4	1.4 ± 0.1	1.1 ± 0.2	0	0.6 ± 0.0	0	3.2 ± 0.2	2.1 ± 0.3	1.9 ± 0.1	2.2 ± 0.3
102	1614	*cis*-Isolongifolanone	0	1.6 ± 0.3	0	0.61 ± 0.04	0	0	0	0	0	0	0.8 ± 0.2	0
103	1617	Junenol	0.5 ± 0.1	0.3 ± 0.3	0	0	0	0	0	0	0	0	0	0
104	1619	1,10-di-*epi*-Cubenol	0	0	1.51 ± 0.03	0	0	0	0	0.8 ± 0.2	0	1.4 ± 0.8	0	0
105	1620	10-*epi*-γ-Eudesmol	0	0	0	0	0	0	0	0	1.1 ± 0.3	0	0	0
106	1630	1-*epi*-Cubenol	1 ± 1	2.3 ± 0.1	0	2.7 ± 0.3	0	0	0	0.7 ± 0.2	2.4 ± 0.3	1.0 ± 0.1	1.67 ± 0.03	0
107	1633	γ-Eudesmol	0	0	0	1.6 ± 0.6	0	0	0	0	0	0	0	0
108	1636	*cis*-Cadin-4-en-7-ol	2 ± 1	0	0	0	0	0	0	0	0	2.8 ± 0.2	0	0
109	1639	*epi*-α-Cadinol	0	0	0	0	0	1.8 ± 0.5	0	0	0	0	0	0
110	1640	*allo*-Aromadendrene epoxide	0	0	0	0	1.1 ± 0.2	0	0	0	0	0	0	0
111	1642	*allo*-Aromadendrene epoxide	0.3 ± 0.1	0	1.1 ± 0.1	0	0	0	0	0	0	0	1.03 ± 0.06	3.5 ± 0.5
112	1643	Caryophylla-4(12),8(13)-dien-5β-ol	0	1 ± 1	2.2 ± 0.2	0	0	0	0	0	0	0	0	0
113	1644	*epi*-α-Muurolol	0.9 ± 0.5	4 ± 2	1.4 ± 0.1	3.6 ± 0.3	0.8 ± 0.1	0	2.2 ± 0.4	3.9 ± 0.9	4.2 ± 1.3	3.2 ± 0.3	1.4 ± 0.1	0
114	1645	α-Muurolol	1.0 ± 0.7	1.8 ± 0.2	1.3 ± 0.1	2.1 ± 0.3	0	0	0.9 ± 0.4	0.6 ± 0.6	1.9 ± 0.2	1.9 ± 0.1	1.3 ± 0.1	0
115	1646	Cubenol	0	0	0	0	0	0	0	0	1.5 ± 1.3	0	0	0
116	1650	β-Eudesmol	0	1.3 ± 0.5	1.1 ± 0.1	1.6 ± 0.2	0.9 ± 0.3	0	0	0	2.0 ± 0.1	1.8 ± 0.1	0	1.3 ± 0.2
117	1653	α-Cadinol	1.6 ± 0.9	6.4 ± 0.7	0	0	1.6 ± 1.4	1.8 ± 0.3	2.0 ± 1.0	3.3 ± 0.8	3.4 ± 0.3	3.6 ± 0.1	1.9 ± 0.1	1.6 ± 0.2
118	1655	*allo*-himachalol	0	0	0	0	0	1.1 ± 0.1	0	0	0	0	0	0
119	1659	Selin-11-en-4α-ol	0	0	0	0	0	0	0	0	0	0.71 ± 0.04	0	0
120	1665	14-hydroxy-(*Z)*-Caryophyllene	0.5 ± 0.4	0	2.3 ± 0.1	0	0	0	1.6 ± 0.2	0.1 ± 0.1	2.2 ± 2.1	2 ± 1	0	0.9 ± 0.1
121	1668	(*Z*)-α-Santalol	0	3.5 ± 0.7	4.3 ± 0.7	1.2 ± 0.2	0	0	0	0	0.8 ± 1.4	1 ± 1	1.43 ± 0.02	0
122	1671	Guaia-3,10(14)-dien-11-ol	0	0	0	0	0	0	0	0	0	0	0	1.1 ± 0.3
123	1674	Khusinol	1.4 ± 1.1	0	1.1 ± 0.1	0	0	0	0	0	0	0	0	0
124	1680	Elemol acetate	0	0	0	0	0	0	0	0	0	0	0.8 ± 0.1	0
125	1682	Germacra-4(15),5,10(14)-trien-1α-ol	0.7 ± 0.3	1.4 ± 0.1	0.9 ± 0.3	0	0	0.35 ± 0.04	0	0.7 ± 0.4	3.3 ± 0.6	2.1 ± 0.2	0	0.4 ± 0.1
126	1689	Eudesma-4(15),7-dien-1β-ol	0.6 ± 0.1	0	0	0.9 ± 0.2	0	0	0.7 ± 0.2	0.5 ± 0.2	1.2 ± 0.2	0	1.2 ± 0.0	0
127	1690	Shyobunol	0	0	0	0	0	0	0	0	1.7 ± 0.2	0	0	0
128	1705	Amorpha-4,9-dien-2-ol	0.9 ± 0.5	0	0	0	0	0	0	0	0.6 ± 0.3	0.2 ± 0.2	0.6 ± 0.1	0.6 ± 0.1
129	1712	*cis*-Thujopsenal	0	0	0	0	0	0	0	0	0.9 ± 0.2	0	0.6 ± 0.1	0
130	1714	Longifolol	0.3 ± 0.3	0	0	0	0	0	0	0	0	0	0	0
131	1733	Curcumenol	0	0	0	0	0	0	0	0	0	0.4 ± 0.1	0	0
132	1737	Isobicyclogermacrenal	0	0.7 ± 0.3	0	0	0	0	0	0	1.4 ± 0.1	1.1 ± 0.1	0	0
133	1743	γ-Costol	0	0	0	0	0	0	0	0	0	0	0.6 ± 0.1	1.4 ± 0.2
134	1751	Cyclocolorenone	0	0	0	0	0	0	0	0	0.8 ± 0.2	0	0.7 ± 0.2	0
135	1752	β-Acoradienol	0	0.5 ± 0.4	0	0	0	0	0	0	0	0	0	0
136	1774	14-oxy-α-Muurolene	0.5 ± 0.5	0	0	0	0	0	0	0	0	0	0	0
137	1777	Squamulosone	0	0.7 ± 0.2	0.9 ± 0.1	0	0	0	0	0	1.0 ± 0.3	0.5 ± 0.1	0	0.4 ± 0.4
138	1779	α-Costol	0.4 ± 0.4	0	0	0	0	0	0	0	0	0	0	0

AI: Arithmetical retention indices calculated using Van den Dool and Kratz [26] equation with *n*-alkanes on a SH-Rtx-5MS capillary column. ^b^ Relative content values are shown as the average of three determinations ± standard deviation.

**Table 2:** Antimicrobial activity (mg/mL) of the essential oils from leaves of *Baccharis uncinella*, *B. retusa* and *B. calvescens*.

Samples	*S. aureus*	*E. coli*	*P. aeruginosa*	*S. choleraesuis*	*C. albicans*	*B. subtilis*	*S. epidermidis*
*Baccharis uncinella*							
Spring	0.062	0.125	*	0.125	0.062	0.125	*
Summer	0.125	0.125	*	0.25	0.062	0.062	*
Autumn	0.125	0.125	*	0.25	0.125	0.125	*
Winter	0.125	0.25	*	0.50	0.25	0.125	*
*Baccharis retusa*							
Spring	0.125	0.25	*	0.50	0.25	0.125	*
Summer	0.125	0.25	*	0.50	0.50	0.125	*
Autumn	0.125	0.25	*	0.50	0.50	0.125	*
*Baccharis calvescens*							
Spring	0.125	0.25	*	0.50	0.50	0.125	*
Summer	0.25	0.25	*	0.50	0.125	0.125	*
Autumn	0.25	0.50	*	0.50	1.0	0.125	*
*Thymus vulgaris* [34]	1.0	*	*	0.6	2.0	0.5	*
Chloramphenicol	0.008	0.004	0.062	0.004		0.004	0.008
Nystatin					0.002		

* MIC > 2.0 mg/mL.

**Table 3:** Anti-proliferative activity, expressed as the concentration required to elicit total cell growth inhibition (TGI, µg/mL), of doxorubicin (positive control) and the essential oils of *Baccharis uncinella*, *B. retusa* and *B. calvescens* aerial parts collected in different seasons during a year.

Samples ^b^	TGI (µg/mL) ^a^									MeanTGI	TGI (µg/mL) ^a^
	U251	UACC-62	MCF-7	NCI-ADR/Res	786-0	NCI-H460	OVCAR-03	HT29	K562		HaCaT
Doxorubicin	10 *	0.35 ± 0.06	4 *	16 *	0.8 ± 0.1	0.17 ± 0.09	2 ± 1	>25	1.5 *	> 6.6	1.9 *
*Baccharis uncinella*											
Autumn	56 ± 2	62 ± 11	60 ± 2	73 ± 13	67 ± 4	61 ± 9	40 ± 4	72 ± 5	22 ± 1	57.0	57 ± 5
Winter	99 ± 24	82 ± 42	157 ± 24	>250	52 *	112 *	19 *	134 ± 37	52 *	>106.3	83 ± 22
Spring	43 *	44 ± 21	50 ± 18	53 *	38 *	56 *	10 *	50 *	3 ± 1	38.6	34 *
Summer	47 ± 1	41 ± 5	24 ± 5	61 ± 5	49 ± 5	50 ± 2	25 ± 2	28 ± 3	19 ± 7	38.2	69.2 ± 0.3
*Baccharis retusa*											
Autumn	70 ± 29	42 *	58 *	72 *	65 ± 23	48 *	67 ± 11	50 *	8.5 *	53.4	77 *
Winter	30.5 ± 0.8	10 ± 3	31 ± 2	44 ± 7	38 ± 3	25 ± 3	17 *	28 ± 4	7.2 ± 0.2	25.6	29 ± 3
Spring	55 ± 11	46 ± 19	56 ± 7	58 ± 4	70 ± 3	57 ± 13	4 *	56 ± 20	18 ± 6	46.7	60 ± 3
Summer	37 *	17 *	9 *	31 *	17 *	23 *	>250	15 *	2 *	44.6	36 *
*Baccharis calvescens*											
Autumn	15 *	15 ± 3	13 ± 2	34 ± 7	16 ± 7	16 ± 8	16 ± 7	20 ± 4	n.t.	18.1	n.t.
Winter	14 ± 2	8 ± 2	1.3 ± 0.4	33 *	15 ± 7	9 ± 1	2.4 ± 0.1	6 ± 3	n.t.	11.1	n.t.
Spring	6.6 ± 0.9	4 ± 1	<0.25	28 *	10 ± 3	6 ± 1	<0.25	4 ± 1	n.t.	<7.4	n.t.
Summer	47 ± 2	41 ± 6	24 ± 8	61 ± 7	49 ± 7	50 ± 2	25 ± 2	46 ± 2	n.t.	42.9	n.t.

(^a^) Results expressed as the concentration of samples required to inhibit cell proliferation by 100% ± standard error, calculated by sigmoidal regression (Origin 8.0 software); (*) approximate value (standard error greater than the calculated effective concentration); mean TGI = arithmetic mean of TGI values observed for tumor cell lines. (^b^) Samples: doxorubicin (positive control; 0.025–25 µg/mL); essential oils of the *Baccharis uncinella*, *B. retusa* and *B. calvescens* aerial parts collected in Autumn (27 May 2015), Winter (25 August 2014), Spring (17 November 2014) and Summer (16 January 2015)—seasons named according to Southern Hemisphere. Human tumor cell lines: U251 (glioblastoma); UACC-62 (melanoma); MCF-7 (breast adenocarcinoma); NCI-ADR/Res (ovarian multidrug-resistant adenocarcinoma); 786-0 (kidney, adenocarcinoma); NCI-H460 (lung, non-small cell carcinoma); OVCAR-03 (ovarian, adenocarcinoma); HT-29 (colorectal adenocarcinoma); K562 (chronic myelogenous leukemia). Human non-tumor cell line: HaCaT (immortalized keratinocytes).

## Data Availability

The data presented in this study are available upon request to the corresponding author.
